# Hyperglycaemic crises in adults with diabetes: a consensus report

**DOI:** 10.1007/s00125-024-06183-8

**Published:** 2024-06-22

**Authors:** Guillermo E. Umpierrez, Georgia M. Davis, Nuha A. ElSayed, Gian Paolo Fadini, Rodolfo J. Galindo, Irl B. Hirsch, David C. Klonoff, Rozalina G. McCoy, Shivani Misra, Robert A. Gabbay, Raveendhara R. Bannuru, Ketan K. Dhatariya

**Affiliations:** 1 Division of Endocrinology, Metabolism, and Lipids, Department of Medicine, Emory University School of Medicine, Atlanta, GA USA; 2 American Diabetes Association, Arlington, VA USA; 3 Department of Medicine, Harvard Medical School, Boston, MA USA; 4 Department of Medicine, University of Padua, Padua, Italy; 5 Veneto Institute of Molecular Medicine, Padua, Italy; 6 Division of Endocrinology, Diabetes and Metabolism, Department of Medicine, University of Miami Miller School of Medicine, Miami, FL USA; 7 Division of Metabolism, Endocrinology, and Nutrition, Department of Medicine, University of Washington, Seattle, WA USA; 8 Diabetes Research Institute, Mills-Peninsula Medical Center, San Mateo, CA USA; 9 Division of Endocrinology, Diabetes and Nutrition, Department of Medicine, University of Maryland School of Medicine, Baltimore, MD USA; 10 University of Maryland Institute for Health Computing, Bethesda, MD USA; 11 Division of Metabolism, Digestion & Reproduction, Imperial College London, London, UK; 12 Department of Diabetes & Endocrinology, Imperial College Healthcare NHS Trust, London, UK; 13 Elsie Bertram Diabetes Centre, Norfolk and Norwich University Hospitals NHS Foundation Trust, Norwich, UK; 14 Department of Medicine, Norwich Medical School, University of East Anglia, Norwich, UK

**Keywords:** Aceto-acetate, Acidosis, Anion gap, B-hydroxybutyrate, Diabetic ketoacidosis, Hyperglycaemia, Hyperglycaemic crises, Hypoglycaemia, Ketones, Ketonuria

## Abstract

The American Diabetes Association (ADA), European Association for the Study of Diabetes (EASD), Joint British Diabetes Societies for Inpatient Care (JBDS), American Association of Clinical Endocrinology (AACE) and Diabetes Technology Society (DTS) convened a panel of internists and diabetologists to update the ADA consensus statement on hyperglycaemic crises in adults with diabetes, published in 2001 and last updated in 2009. The objective of this consensus report is to provide up-to-date knowledge about the epidemiology, pathophysiology, clinical presentation, and recommendations for the diagnosis, treatment and prevention of diabetic ketoacidosis (DKA) and hyperglycaemic hyperosmolar state (HHS) in adults. A systematic examination of publications since 2009 informed new recommendations. The target audience is the full spectrum of diabetes healthcare professionals and individuals with diabetes.

**Supplementary Information:**

The online version contains a slideset of the figures for download available at 10.1007/s00125-024-06183-8.

## Introduction

Diabetic ketoacidosis (DKA) and the hyperglycaemic hyperosmolar state (HHS) are the two most serious, acute and life-threatening hyperglycaemic emergencies in individuals with type 1 diabetes and type 2 diabetes [1–3]. Global reports clearly show an increase in the number of DKA and HHS admissions during the past decade, with recent data reporting a 55% increase in the rate of DKA hospitalisations, especially in adults aged <45 years [4–6]. DKA is characterised by the triad of hyperglycaemia, increased ketone concentration in the blood and/or urine, and metabolic acidosis, while HHS is characterised by severe hyperglycaemia, hyperosmolality, and dehydration in the absence of significant ketosis or acidosis. The metabolic derangements in DKA result from the combination of absolute or relative insulin deficiency (levels insufficient to suppress gluconeogenesis and ketone production) and elevation of counterregulatory hormones (glucagon, adrenaline [epinephrine], noradrenaline [norepinephrine], cortisol and growth hormone) [1, 3, 7]. In HHS, there is a residual amount of insulin secretion that minimises ketosis but does not control hyperglycaemia [1, 3].

Both DKA and HHS can occur at any age in people with type 1 diabetes, type 2 diabetes or any other type of diabetes. DKA is more common in young people with type 1 diabetes, and HHS is more frequently reported in older adults with type 2 diabetes. Although any acute illness or physiological stress can precipitate DKA and HHS, the most frequent causes are infection, particularly urinary tract infections and pneumonia, and the omission of insulin therapy. In recent years, sodium–glucose cotransporter 2 (SGLT2) inhibitors have been found to increase the risk of DKA, most often when used in type 1 diabetes but also in type 2 diabetes [2]. The incidence of both DKA and HHS was reported to have increased during the coronavirus disease-2019 (COVID-19) pandemic [8, 9]. Early diagnosis and management of DKA and HHS are essential to improve outcomes. The mainstays of treatment of DKA and HHS are fluid replacement, insulin therapy, electrolyte repletion and treatment of underlying precipitating events. Appropriate treatment has reduced mortality owing to DKA to <1%; however, mortality has remained five- to tenfold higher in individuals with HHS [1, 10].

The objective of this consensus report is to provide up-to-date knowledge about the epidemiology, pathophysiology, clinical presentation, and recommendations for the diagnosis, treatment and prevention of DKA and HHS in adults. The target audience is the full spectrum of diabetes healthcare professionals and individuals with diabetes.

## Research design and methods

This consensus report is an update of the American Diabetes Association (ADA) consensus statement on hyperglycaemic crises in adults with diabetes, published in 2001 and last updated in 2009 [11, 12]. The ADA convened a panel of internists and diabetologists representing the ADA, European Association for the Study of Diabetes (EASD), Joint British Diabetes Societies for Inpatient Care (JBDS), American Association of Clinical Endocrinology (AACE) and Diabetes Technology Society (DTS).

At the beginning of the writing process, all members of the expert panel participated in a day-long virtual meeting and agreed on the direction for this consensus report, the methodology and rigour to be followed for this report, and the established writing teams to author the various sections of the report. The writing group, with the help of a methodologist, conducted comprehensive literature searches in PubMed using medical subject headings to identify human studies published in English between 1 January 2009 and 1 June 2023. To identify contemporary evidence, they included information from observational studies, randomised controlled trials and systematic reviews.

Monthly calls were held between October 2022 and September 2023, with additional e-mail and web-based collaboration. One in-person meeting was conducted to provide organisation to the process, establish the review process, reach consensus on the content and key definitions, and discuss the recommendations. Once the draft was completed, the structured peer review process was implemented, and the report was sent to external peer reviewers and respective committees of all the contributing organisations. A final draft was completed and submitted to all five organisations for final review and approval. The guidance represents the panel's collective analysis, evaluation and expert opinion.

Questions related to clinical practice provide the framework for this update on hyperglycaemic crises in adults. This update includes eight sections that cover new evidence about epidemiology, pathogenesis, diagnostic criteria, recommended treatment, complications during treatment, management in special populations, prevention and priority areas for future research.

## Section 1. What are recent global trends in epidemiology and outcomes?

Nearly 1% of all hospitalisations in people with diabetes are for hyperglycaemic crises. However, estimates vary widely among studies because of different populations, settings, types of events captured and methods of event ascertainment. In a US-based study, 38% of hospital admissions for hyperglycaemic crises were for DKA, 35% for HHS and 27% for mixed DKA/HHS [10]. Most DKA events occur in young adults aged 18–44 years (61.7%) with type 1 diabetes (70.6%), while HHS events are more common among middle-aged adults 45–64 years (47.5%) with type 2 diabetes (88.1%) [13]. Additionally, several studies have revealed that over half of Black/African American and Hispanic/Latino adults with newly diagnosed diabetes presenting with unprovoked DKA have type 2 diabetes [14–16]. The clinical presentation in such cases is acute, as in classical DKA observed in people with type 1 diabetes; however, after immediate stabilisation and a short course of insulin therapy, prolonged near-euglycaemia is often possible because of restoration of pancreatic beta cell function and insulin sensitivity, with gradual cessation of insulin treatment and maintenance of glycaemic goals with medical nutrition therapy and non-insulin agents [4]. Such individuals often have clinical and metabolic features of type 2 diabetes, including high rates of obesity, a strong family history of diabetes, a measurable pancreatic insulin reserve, the absence of autoimmune markers of beta cell destruction, and the ability to discontinue insulin therapy during follow-up [14, 17]. This presentation of diabetes has been referred to in the literature as atypical diabetes or ketosis-prone type 2 diabetes [14, 17].

Epidemiological studies conducted in the USA and Europe over the past decade have revealed a concerning rise in the rate of hyperglycaemic emergencies in adults with both type 1 diabetes and type 2 diabetes [4–6, 13, 18–21]. This represents a marked departure from the previously observed improvements seen between 2000 and 2009 [6]. During the first decade of the 21st century, reported incidence rates of DKA in adults with type 1 diabetes in Europe, the USA and Israel have varied between 0 and 56 events per 1000 person-years, although one study conducted in China between 2010 and 2012 reported an outlying rate of 263 per 1000 person-years [22]. No population-level data are available for HHS or mixed DKA/HHS episodes, but some studies grouped all hyperglycaemic crises together, as it can be challenging to reliably classify events using administrative data such as hospitalisation databases that many studies rely on. Among people with type 1 diabetes, most recent data suggest hyperglycaemic crisis rates of up to 44.5–82.6 per 1000 person-years [5, 21] and among people with type 2 diabetes up to 3.2 per 1000 person-years [5].

A substantial proportion of individuals hospitalised with DKA experience recurrent episodes [23], underscoring the importance of engaging patients experiencing these events to identify triggers and prevent recurrence. In a US-based study conducted between 2006 and 2012 in Chicago, IL, 21.6% of people hospitalised for DKA had more than one episode over 6 years, with 5.8% of individuals accounting for 26.3% of DKA hospitalisations [23]. Similarly, analysis of inpatient data from the UK in 2014 revealed that 33.7% of people admitted with DKA had at least one episode of DKA in the prior year [24]. In general, the all-cause readmission rate after episodes of DKA or hyperglycaemic crises in general ranges between 10% and 20%, with 40–65% of these readmissions being for recurrent hyperglycaemic crises (the remainder are for other causes, including occasionally for severe hypoglycaemia), mostly occurring within 2 weeks of discharge from the prior DKA episode [25–27].

### Morbidity and mortality

Hyperglycaemic crises are associated with substantial morbidity, mortality and costs [28–31]. In the USA, the mean length of stay for patients hospitalised with DKA is 3.0 days among people with type 1 diabetes and 3.7 days among people with type 2 diabetes [32] and has been shortening over time [29]. In the UK, the mean length of stay is generally higher, at 5.6 days [28]. In US-based studies, hospital charges for DKA admissions have ranged from $21,215 to $36,600 per admission, are higher for individuals with type 2 diabetes than for those with type 1 diabetes, and have been rising over time [25, 29, 31–33]. In the UK, costs of DKA admission were estimated at £2064 per hospitalisation [28].

While DKA mortality appeared to be decreasing in studies conducted between 2007 and 2014 [6, 19, 29], these improvements have plateaued in the past decade [4, 21, 34]. Recent estimates reported an inpatient mortality during hospital admission for DKA ranging from 0.20% in type 1 diabetes to 1.04% in type 2 diabetes [6, 32]. Inpatient mortality among people with type 2 diabetes hospitalised for HHS decreased from 1.44% in 2008 to 0.77% in 2018 [20]. Patients with mixed DKA/HHS have higher hospital mortality than those with HHS (adjusted OR 2.7 [95% CI 1.5, 4.9]) or with DKA (adjusted OR 1.8 [95% CI 0.9, 3.6]), with inpatient mortality rates of 8% for mixed DKA/HHS, 5% for HHS and 3% for DKA [10]. In Japan, inpatient mortality has been reported as 3.3–5.7% in DKA admissions, 13.2% in HHS and 5.3% in mixed DKA/HHS admissions [35, 36]. Mortality rates reported in low- and middle-income countries are much higher, potentially because of delayed diagnosis and treatment. Inpatient mortality in DKA admissions has ranged from 26% to 41.3% in sub-Saharan Africa [37], 30% in India [37] and 23.6% in Pakistan [38]. In Jamaica, inpatient mortality has been reported as 6.7% in DKA admissions, 20.3% in HHS and 25% in mixed DKA/HHS admissions [39]. In Nigeria, inpatient mortality has been reported as 2.7% in DKA, 0.9% in HHS and 3.6% in mixed DKA/HHS [40].

People discharged after an episode of DKA have a 1 year age-corrected mortality rate that is 13 times higher than the general population [41]. This is more pronounced among younger individuals (aged 15–39 years), in whom the mortality rate is 49 times higher than the general population [41]. In the USA, all-cause mortality within 30 days of a hyperglycaemic crisis is 0.1% among patients with type 1 diabetes and 2.0% among patients with type 2 diabetes [34]. The 1 year mortality rates were 0.9% and 9.5% in patients with type 1 diabetes and type 2 diabetes, respectively [34]. Compared with patients with a single DKA admission, those with 2–5 admissions have a threefold higher risk of death, while those with six or more admissions have a sixfold higher risk of death [42]. Post-hospital mortality data for HHS are scarce, with one Italian study reporting a 30 day mortality rate after HHS of 16% [43].

### Risk factors

Between 6% and 21% of adults present with DKA as their initial diagnosis of type 1 diabetes [21, 24, 44]. In adults with a known history of diabetes, the most common precipitating factors for DKA include infections, intercurrent illnesses, psychological stress, and omission or insufficient use of insulin therapy, as described in Table 1 [24, 27, 28, 30, 38, 44–52]. Worldwide, infection is the most common precipitating factor for DKA, occurring in 14–58% of cases [3, 24]. Other acute conditions that may precipitate DKA include stroke, alcohol and substance use, pancreatitis, pulmonary embolism, myocardial infarction and trauma [1, 53–56].

**Table 1:** Precipitating causes of DKA in adults by region

Region	New-onset diabetes	Infection	Insulin omission	Other	Unknown
Australia	5.7	28.6	40	25.7	NR
Brazil	12.2	25	39	15	8.8
China	NR	39.2	24	10.9	25.9
Indonesia	3.3	58.3	13.3	17.1	8
South Korea	NR	25.3	32.7	11.2	30.8
Nigeria	NR	32.5	27.5	4.8	34.6
Spain	12.8	33.2	30.7	23.3	NR
Syria	NR	47.8	23.5	7.8	20.9
Taiwan	18.2	31.7	27.7	6.2	16.2
UK	6.1	44.6	19.7	10.9	18.7
USA	17.2–23.8	14.0–16.0	41.0–59.6	9.7–18.0	3.0–4.2

Data are %. Adapted from Dhatariya et al [3]

NR, not reported

The omission of insulin therapy, often in the setting of psychological and socioeconomic factors, is a major cause of DKA, particularly among adults with type 1 diabetes living in socioeconomically deprived areas [1, 24, 48, 54, 57]. A study assessing the clinical, socioeconomic and psychological factors associated with DKA recurrence in urban patients from racial and ethnic minority backgrounds found discontinuation of insulin therapy to account for more than two-thirds of all DKA admissions [48].

Factors associated with a higher risk of hyperglycaemic crisis in people with type 1 diabetes include younger age, prior history of hyperglycaemic and hypoglycaemic crises, presence of kidney disease, neuropathy, depression, smoking, alcohol and substance abuse, high HbA_1c_ and social determinants of health (SDOH) [1, 6, 7, 16, 55, 58]. In people with type 2 diabetes, risk factors include younger age, prior history of hyperglycaemic or hypoglycaemic crises, presence of comorbidities (both diabetes-related and unrelated), and elevated HbA_1c_ and SDOH [7, 16, 42, 48]. Multiple studies have suggested that low income, area-level deprivation, housing insecurity, and lack of insurance or presence of underinsurance (e.g., having a high deductible health plan or Medicaid coverage in the USA) lead to increased risk of DKA and HHS [7, 10, 16, 31, 33, 59, 60], with approximately 40% of hyperglycaemic crises occurring in lower-income and underserved populations [13, 61]. Food insecurity is also associated with triple the rate of DKA in youth and young adults with type 2 diabetes [62]. In addition, SDOH and mental health conditions are the strongest factors associated with recurrent DKA [23, 25, 31, 42].

People with diabetes who have a history of DKA (compared with those without such a history) have been reported to have a significantly higher prevalence of mental health disorders such as depression, diabetes distress, substance abuse, psychoses and bipolar disorder [63]. Psychological comorbidities, including eating disorders, have been reported in recurrent episodes of DKA in young women [64, 65]. Depression and psychological comorbidities have a correlation with decreased blood glucose monitoring and treatment engagement, which are associated with an increased risk of hospitalisation for hyperglycaemic crises [66]. In addition, observational studies have reported that people with type 1 diabetes and a history of DKA have an increased prevalence of depression and risk of hospitalisation for a suicide attempt, with the highest risk of suicide attempt in the 12 months following the DKA episode [67, 68]. Importantly, the relationship between mental health conditions and hyperglycaemic crises may be bidirectional, and all individuals experiencing hyperglycaemic crises should be screened for mental health concerns. The Patient Health Questionnaire (PHQ-9) is the most used and validated screening test for depression in people with diabetes, with a high sensitivity and specificity [69]. Importantly, symptoms associated with hyperglycaemia may complicate screening because they may be mistaken for symptoms of depression (e.g., fatigue, hypersomnia, psychomotor slowing). In addition, screening for diabetes distress is indicated using the T1-Diabetes Distress Assessment System (T1-DDAS) to assess the degree of emotional burden related to diagnosis and management of diabetes, particularly type 1 diabetes, that can influence management behaviours and clinical outcomes [70].

Recent studies have shown mixed results regarding the risk of DKA with insulin pump therapy. Some studies have shown improved glycaemic goals and a reduced risk of both DKA and severe hypoglycaemia in insulin pump users [71, 72]. However, other studies have shown higher rates of DKA with insulin pumps in type 1 diabetes [73, 74]. In pump users presenting with DKA, the most common precipitating factors are management error and underlying infection; these are more common precipitating causes than device malfunction [74]. As insulin pumps increasingly become integrated with continuous glucose monitoring (CGM) in automated insulin delivery systems, these systems may be associated with less DKA and higher rates of attaining glycaemic management goals [75–77]; however, larger studies and real-world data are still needed.

Several studies have reported DKA at the presentation of newly diagnosed type 1 diabetes during or after a COVID-19 infection [9, 78]. The precise mechanisms for new-onset diabetes in people with COVID-19 are not known, but several complex interrelated processes may be involved, including detection of previously undiagnosed diabetes, stress hyperglycaemia, steroid-induced hyperglycaemia, and direct or indirect effects of severe acute respiratory syndrome coronavirus 2 on the beta cell [8, 9]. Rates of DKA during the COVID-19 pandemic increased primarily among individuals with newly diagnosed diabetes and preexisting type 2 diabetes [79, 80]. While rates of DKA decreased among people with preexisting type 1 diabetes in the UK, they increased among people with type 1 diabetes in the USA [79, 81]. Older adults from racial and ethnic minority backgrounds experienced the greatest rise in DKA events [79, 81].

Some drug classes can affect carbohydrate metabolism and precipitate the development of DKA and HHS [82]. Glucocorticoids may precipitate acute and sustained hyperglycaemia by countering insulin action [83, 84]. Antipsychotic medications may also raise DKA risk, although the precise mechanism is uncertain [85]. Approximately 1–2% of patients receiving checkpoint inhibitors develop new-onset autoimmune diabetes [86], characterised by rapid onset of hyperglycaemia, swift progression of endogenous insulin deficiency, and a high risk of DKA or severe hyperglycaemia if not detected and treated promptly with insulin therapy [87, 88]. A recent systematic review of 278 patients with checkpoint inhibitor-associated autoimmune diabetes reported that DKA was present at diagnosis in 69.7%, while hyperglycaemia without acidosis was present in the remainder [89].

DKA risk is also increased with SGLT2 inhibitors in adults with type 1 diabetes [90, 91] and insulin-deficient type 2 diabetes [92]. SGLT2 inhibitor-associated DKA occurs in approximately 4% of people with type 1 diabetes; the risk can be 5–17 times higher than in people with type 1 diabetes not treated with SGLT2 inhibitors [90]. In contrast, observational studies and randomised controlled trials have shown that DKA is uncommon in people with type 2 diabetes treated with SGLT2 inhibitors, with an estimated incidence of 0.6–4.9 events per 1000 patient-years [93]. A meta-analysis of four randomised controlled trials found the relative risk (RR) of DKA in participants with type 2 diabetes treated with SGLT2 inhibitors vs placebo or active comparator arm to be 2.46 (95% CI 1.16, 5.21), while a meta-analysis of five observational studies found the RR to be 1.74 (95% CI 1.07, 2.83) [94]. Risk factors for DKA in individuals with type 2 diabetes treated with SGLT2 inhibitors include very-low-carbohydrate diets and prolonged fasting, dehydration, excessive alcohol intake and the presence of autoimmunity, in addition to typical precipitating factors [94, 95]. Notably, in one series, 35% of people treated with SGLT2 inhibitors presenting with DKA had glucose levels <11.1 mmol/l (200 mg/dl) [96], and in another series, 71% of people treated with SGLT2 inhibitors presenting with DKA had glucose levels ≤13.9 mmol/l (250 mg/dl) [97].

Volume depletion is a primary driver of HHS, which commonly occurs in older adults with above-target glucose levels who are at particularly high risk for developing dehydration because of polyuria, age-related impairment of thirst mechanisms, and limited access to fluids [7, 98]. Infection is the major precipitating factor in 30–60% of patients with HHS, with urinary tract infections and pneumonia being the most common [99]. Other common precipitating causes of HHS include acute cerebrovascular events, acute myocardial infarction, surgery, acute pancreatitis, and the use of drugs that affect carbohydrate metabolism by decreasing insulin release or activity. These include corticosteroids, sympathomimetic agents and antipsychotic drugs [1, 99].

## Section 2. What is the pathogenesis of hyperglycaemic crises?

**Fig. 1:**
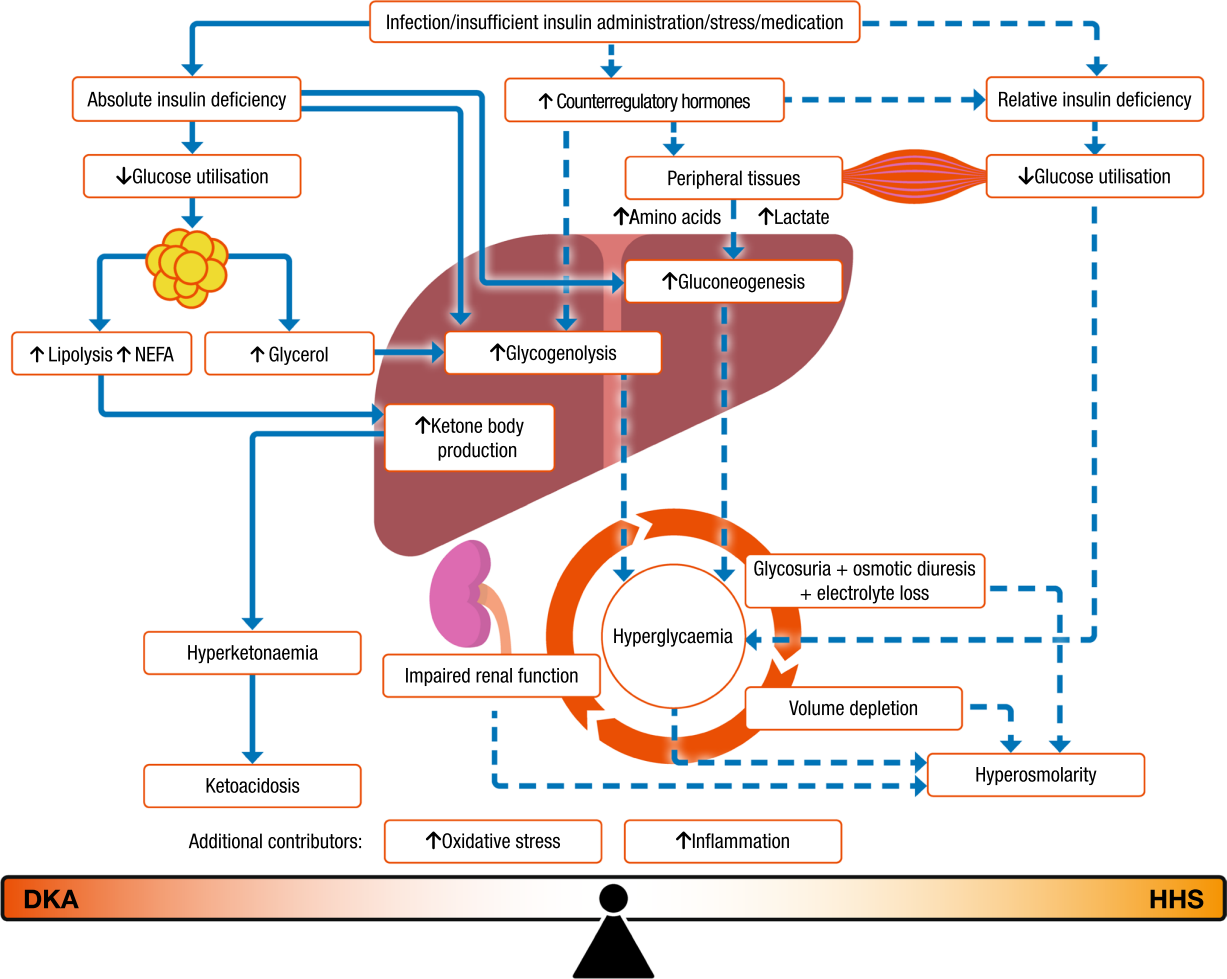
Pathogenesis of DKA and HHS. This figure is available as part of a downloadable slideset

The key difference between DKA and HHS is the degree of insulin insufficiency. The pathogenesis of these two diseases is presented in Fig. 1. DKA is characterised by severe insulin deficiency and a rise in concentrations of counterregulatory hormones (glucagon, cortisol, adrenaline and growth hormones) [1, 3, 7]. The resulting changes in the insulin/glucagon ratio lead to increased gluconeogenesis, accelerated glycogenolysis, and impaired glucose utilisation by peripheral tissues. The combination of insulin deficiency and increased counterregulatory hormones results in the release of NEFAs from adipose tissues (lipolysis), leading to unrestrained hepatic fatty acid oxidation and the production of excess ketone bodies with resulting ketonaemia and metabolic acidosis [3].

In HHS, compared with DKA, there is less severe insulin deficiency and, therefore, sufficient insulin to prevent ketogenesis but not enough to prevent hyperglycaemia, due to increased hepatic glucose production and decreased glucose utilisation by peripheral tissues. Hyperglycaemia leads to an osmotic diuresis, leading to volume depletion and haemoconcentration. If fluid intake is not maintained, then this can lead to a hyperosmolar state, renal impairment and, ultimately, a decline in cognitive function (Fig. 1).

Hyperglycaemia in people with hyperglycaemic crises is associated with a severe inflammatory state characterised by an elevation of proinflammatory cytokines (tumour necrosis factor-α, and interleukin-1, -6 and -8), C-reactive protein, reactive oxygen species, and lipid peroxidation biomarkers even in the absence of obvious infection or cardiovascular pathology [100]. All these measurements return to near-normal values within 24 h following correction of hyperglycaemia with insulin therapy and hydration.

## Section 3. What are the diagnostic criteria of DKA and HHS?

### Diagnostic criteria for DKA

**Fig. 2:**
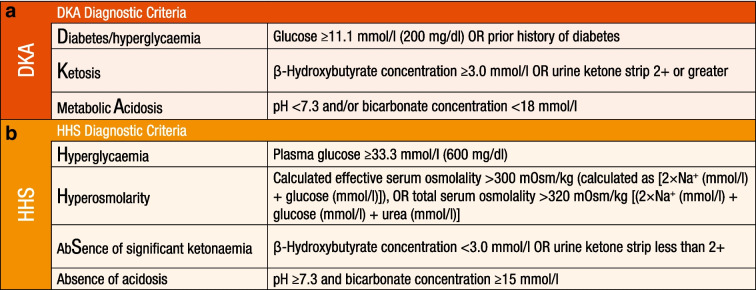
The diagnosis criteria of (**a**) DKA and (**b**) HHS. This figure is available as part of a downloadable slideset

The diagnosis of DKA should be based on the three criteria described in Fig. 2a. All three components must be present to make this diagnosis. In this consensus report, we have defined hyperglycaemia as a diagnostic criterion for DKA from >13.9 mmol/l (250 mg/dl) to either a glucose value of ≥11.1 mmol/l (200 mg/dl) or a prior history of diabetes irrespective of the presenting glucose value. Hyperglycaemia and/or diabetes must be accompanied by two additional criteria—elevated ketones and metabolic acidosis—for the diagnosis of DKA to be established. Although hyperglycaemia remains a key diagnostic criterion of DKA, a wide range of plasma glucose concentrations can be present on admission. Approximately 10% of patients with DKA present with euglycaemic DKA, which is defined as plasma glucose levels <11.1 mmol/l (200 mg/dl) in the presence of ketosis and metabolic acidosis criteria of DKA described in Fig. 2 [91, 101, 102]. Euglycaemic DKA can be caused by a variety of factors, including exogenous insulin injection, reduced food intake, pregnancy, or impaired gluconeogenesis due to alcohol use, liver failure and/or SGLT2 inhibitor therapy [103, 104]. In recent years, the use of SGLT2 inhibitors in those with type 1 diabetes and type 2 diabetes has accounted for the majority of cases of euglycaemic DKA [105–107]. In recognition of the wider range of glucose levels at presentation with DKA, the criteria for diagnosis of DKA have been changed to encompass a lower glucose value of >11.1 mmol/l (200 mg/dl) and a prior history of diabetes (irrespective of the glucose level) [2].

The key diagnostic feature in DKA is the elevation of the circulating total ketone body concentration. Assessment of ketonaemia can be performed semiquantitatively by the nitroprusside reaction in urine or serum, which measures acetoacetic acid (but not β-hydroxybutyrate, the main ketoacid produced in DKA), or quantitatively by direct measurement of β-hydroxybutyrate in blood from capillary point-of-care testing (POCT) or in the hospital laboratory [3]. Both types of ketones have similar diagnostic sensitivity, but measuring β-hydroxybutyrate in blood is more specific for detecting DKA than measuring acetoacetate in urine [108].

Reliance on urine ketone testing can underestimate the severity of ketonaemia early in the course of DKA because of a lag in the formation of acetoacetate, and conversely overestimate its severity later in the course of DKA when β-hydroxybutyrate is being cleared and converted into acetoacetate [3]. In addition, several sulfhydryl drugs (e.g., captopril) and medications such as valproate can give false-positive nitroprusside urine tests [109]. Thus, for diagnosis and monitoring of the response to therapy, we recommend direct measurement of venous or capillary β-hydroxybutyrate, which is the main ketoacid in DKA [3, 108]. Blood concentrations of β-hydroxybutyrate ≥3.0 mmol/l correlate well with acid–base changes, with >90% sensitivity and specificity for the diagnosis of DKA [1, 2, 12]. β-Hydroxybutyrate measurement can be performed on serum samples using laboratory analysis or capillary blood samples using handheld POCT meters with similar precision in quantifying β-hydroxybutyrate [3, 108]. Compared with a laboratory measurement, the convenience of testing and rapidity of results from POCT can reduce the time for assessment, duration of admission and time to recovery from DKA [2, 12, 110]. A systematic review of nine studies on the accuracy of capillary β-hydroxybutyrate measurement for identifying DKA, compared with multiple other analytical and clinical tests, reported high sensitivity, specificity, and positive and negative predictive values [111]. However, there is concern about how accurate POCT instruments are compared with laboratory instruments for measuring β-hydroxybutyrate levels ≥5 mmol/l [108, 112].

Most people with DKA present with a high anion gap metabolic acidosis. The anion gap is calculated by subtracting the major measured anions (chloride and bicarbonate) from the major measured cation (sodium). An anion gap >12 mmol/l indicates the presence of a high anion gap metabolic acidosis consistent with DKA. However, mixed acid–base disorders are present in about one-third of those presenting with DKA because of hyperglycaemia-induced osmotic diuresis and natriuresis, nausea and vomiting leading to volume contraction and metabolic alkalosis, and a compensatory respiratory alkalosis caused by hyperventilation due to rapid and/or deep breathing (Kussmaul breathing) [113, 114]. In addition, hyperchloraemic normal anion gap acidosis is commonly seen following successful treatment of DKA and may delay transition back to subcutaneous insulin if mistaken for persistent DKA [7, 115]. Although the anion gap is not recommended as a first-line diagnostic or resolution criterion for these reasons, it may still have some utility in resource settings where ketone measurement is unavailable.

The severity of DKA is classified as mild, moderate or severe based on the magnitude of metabolic acidosis (blood pH, serum bicarbonate and ketone levels) and the presence of altered mental status, as presented in Table 2 [12]. This categorisation may be clinically useful for guiding the location where an individual is assigned to receive care (e.g., emergency department, intensive care unit [ICU] or step-down unit) and for identifying patients with mild DKA who are candidates for subcutaneous insulin dosing rather than intravenous insulin infusion [116]. However, not all variables need to be fulfilled to be defined as either mild, moderate or severe, and the admission site and level of care are ultimately a clinical decision.

**Table 2:** DKA classification and suggested level of care by severity: mild, moderate or severe

	Mild DKA	Moderate DKA	Severe DKA
‘D’: history of diabetes or elevated glucose level	Glucose ≥11.1 mmol/l (200 mg/dl)	Glucose ≥11.1 mmol/l (200 mg/dl)	Glucose ≥11.1 mmol/l (200 mg/dl)
‘K’: ketonaemia	β-Hydroxybutyrate 3.0–6.0 mmol/l	β-Hydroxybutyrate 3.0–6.0 mmol/l	β-Hydroxybutyrate >6.0 mmol/l
‘A’: acidosis	• pH >7.25 to <7.30 or bicarbonate 15–18 mmol/l	• pH 7.0–7.25 • Bicarbonate 10 to <15 mmol/l	• pH <7.0 • Bicarbonate <10 mmol/l
Mental status	Alert	Alert/drowsy	Stupor/coma
Suggested level of care	Regular or observation nursing unit	Step-down unit or intermediate care unit	ICU

Not all variables need to be fulfilled to be defined as either mild, moderate or severe, and the admission site and level of care are ultimately a clinical decision

### Diagnostic criteria for HHS

HHS is a state of significant hyperglycaemia and hyperosmolality in the absence of severe ketonaemia and metabolic acidosis. The diagnosis of HHS should be based on the four criteria presented in Fig. 2b. All four components must be present to make the diagnosis [12, 117].

Clinical overlap between DKA and HHS has been reported in more than one-third of people with hyperglycaemic crises [50]. Although most people with HHS have an admission pH ≥7.30 and a bicarbonate level ≥18 mmol/l, mild ketonaemia may be present.

### Clinical presentation of DKA and HHS

**Fig. 3:**
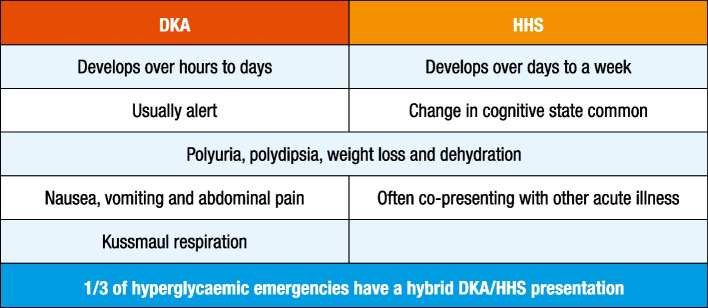
Clinical presentation in patients with DKA and HHS. This figure is available as part of a downloadable slideset

Figure 3 illustrates common clinical features in individuals admitted with DKA and HHS. In DKA, the time between initial symptoms and acute presentation may be hours to a few days, whereas with HHS, it may take days or weeks to develop. Both conditions may present with polyuria, polydipsia, weight loss, vomiting, dehydration and change in cognitive state. The respiratory compensation for metabolic acidosis found in DKA is manifest by Kussmaul breathing, which consists of deep breaths with a fruity odour smell because of the presence of acetone (a breakdown product of the ketone acetoacetic acid) in the breath. Changes in cognitive state are usually present in patients with severe DKA and HHS. Nausea, vomiting and abdominal pain are common in DKA (>50%) but are uncommon in HHS [118]. Caution is needed with patients who present with abdominal pain because the symptoms could be either a result of the DKA or an indication of a precipitating cause of DKA, particularly in the absence of severe metabolic acidosis. Further clinical evaluation is necessary if this complaint is not resolved with the resolution of dehydration and metabolic acidosis.

If DKA or HHS is suspected, initial samples should be taken for glucose, serum electrolytes, venous blood gases, complete blood count, and blood or urine ketone levels. Volume status can be assessed with vital sign parameters. Tachycardia and hypotension correlate with severe hypovolaemia. However, some patients can maintain haemodynamic stability and intravascular volume because of the hypertonicity associated with hyperglycaemia and the subsequent movement of intracellular water into the extracellular space. Patients should be examined for signs of infection, ischaemia and other potential precipitants of a hyperglycaemic crisis. In addition, an electrocardiogram should be performed to assess for evidence of biochemically induced repolarisation abnormalities, such as peaked T waves from hyperkalaemia and ischaemia.

It is important to consider the differential diagnosis of elevated ketones, including starvation ketosis, alcoholic ketoacidosis, and ketosis of pregnancy and hyperemesis [3]. The diagnosis of starvation ketosis is suggested by a history of dietary intake of <2090 kJ/day (500 kcal/day), which is associated with low insulin concentrations, leading to ketone production. People with chronic ethanol use with a recent binge culminating in vomiting and acute starvation may develop ketoacidosis with or without hyperglycaemia [119, 120]. The vomiting of hyperemesis gravidarum leads to excess counterregulatory hormone concentrations, also predisposing to ketone formation.

## Section 4. What is the recommended treatment of DKA and HHS?

**Fig. 4:**
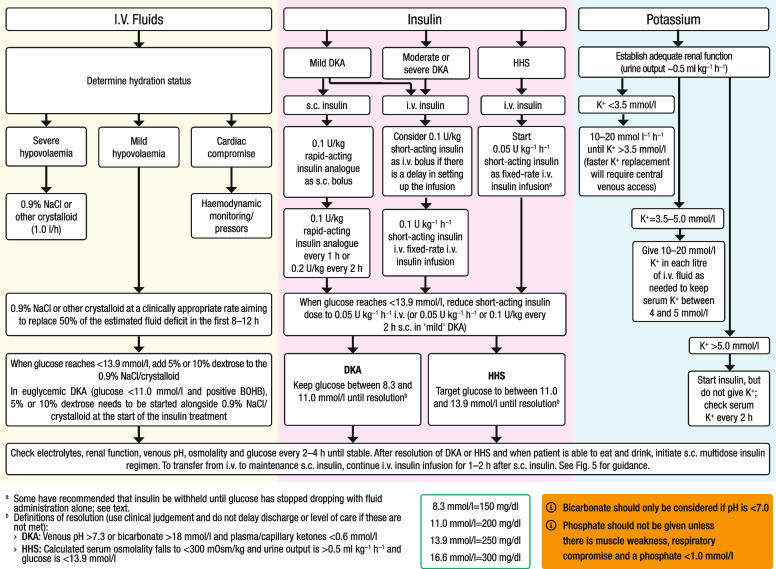
Treatment pathways for DKA and HHS. BOHB, β-hydroxybutyrate. This figure is available as part of a downloadable slideset

DKA and HHS have a similar underlying pathogenesis consisting of insulin deficiency, increased counterregulatory hormones, and loss of fluid and electrolytes. The management of DKA and HHS includes the administration of intravenous fluids, insulin and electrolytes as well as identification and treatment of the precipitating cause. Capillary blood glucose testing should be performed during treatment every 1–2 h using a hospital-calibrated glucose meter, and blood should be drawn every 4 h for determination of electrolytes, phosphate, creatinine, β-hydroxybutyrate and venous pH until resolution of DKA. In patients with HHS, in addition to measuring glucose, creatinine and electrolytes, serum osmolarity should be measured every 4 h. Treatment pathways for DKA and HHS emphasising intravenous fluids, short-acting insulin and potassium are illustrated in Fig. 4.

Most people with uncomplicated mild or moderate DKA can be treated in the emergency department or a step-down unit if close nursing supervision and monitoring are available [121]. In such patients, several comparisons of treating DKA in the ICU vs step-down and general nursing units have not demonstrated clear differences in mortality rate, length of hospital stay or time to resolution of ketoacidosis. ICU admission in people with mild DKA has also been associated with more laboratory testing and higher hospitalisation costs [122, 123]. In contrast, individuals with severe DKA or HHS, or those with critical illness as the precipitating cause (e.g., myocardial infarction, gastrointestinal bleeding, sepsis) or with altered mental status [1, 3, 12, 124] should be treated in the ICU, as outlined in Table 2.

### Fluid therapy

Initial intravenous fluid resuscitation restores the effective circulating intravascular volume, increases tissue/organ perfusion (which decreases lactate formation), improves renal perfusion (which promotes renal excretion of glucose and ketone bodies), corrects electrolyte deficits and decreases plasma osmolarity. In addition, correction of a fluid deficit improves insulin sensitivity by reducing counterregulatory hormone concentrations [7, 12]. Mean plasma glucose concentrations have been reported to drop by approximately 2.8–3.9 mmol l^−1^ h^−1^ (50–70 mg dl^−1^ h^−1^) solely in response to intravenous fluid administration in the absence of insulin [2]. This rate of decrease may be even more pronounced in HHS.

The fluid choice for initial resuscitation should be determined by local availability, cost and resources. Most clinical guidelines recommend the administration of isotonic saline (0.9% sodium chloride solution) as the initial resuscitation fluid because of its widespread availability, lower cost, and efficacy in restoring circulating volume in clinical studies [2, 12]. While effective, its use in large volumes may be associated with hyperchloraemic normal anion gap metabolic acidosis and prolonged length of ICU and hospital stay [125]. Recent prospective and observational studies and meta-analyses have reported that the administration of balanced crystalloid solutions (e.g., Ringer’s lactate or plasmalyte-148), compared with the administration of the isotonic saline solution, results in faster DKA resolution [125–129], shorter hospital length of stay and less frequent development of hyperchloraemic metabolic acidosis.

In adults with DKA or HHS without renal or cardiac compromise, we recommend starting the administration of isotonic saline or balanced crystalloid solutions at an initial rate of 500–1000 ml/h during the first 2–4 h. After restoration of intravascular volume, the subsequent choice for fluid replacement depends on the state of hydration assessed by blood pressure, heart rate, fluid input–output balance and sodium concentration. Fluid replacement should correct estimated deficits within the first 24–48 h. However, caution should be used when rapidly replacing fluids in those at high risk of fluid overload, including older adults, pregnant individuals, and people with heart or kidney disease or other serious comorbidities.

In patients with DKA, plasma glucose concentrations usually decrease to <13.9 mmol/l (250 mg/dl) within 4–8 h, which is before ketoacidosis resolves [130]. Thus, once the plasma glucose concentration is <13.9 mmol/l (250 mg/dl), replacement fluids should be modified to contain 5–10% dextrose in addition to the 0.9% sodium chloride to prevent hypoglycaemia and allow continued insulin administration until the ketonaemia is corrected [7, 12].

In patients with HHS, the usual time to resolve hyperglycaemia is between 8 and 10 h and the decline should not exceed 5–6.7 mmol l^−1^ h^−1^ (90–120 mg dl^−1^ h^−1^) to prevent cerebral oedema. Similarly, the rate of decline of serum sodium should not exceed 10 mmol/l in 24 h and the rate of fall in osmolality should be no greater than 3.0–8.0 mOsm kg^−1^ h^−1^ to minimise the risk of neurological complications [117]. Initial fluid replacement will lower the glucose concentration and osmolality, causing a shift of water into the intracellular space, which may result in a rise in serum sodium (a reduction of 5.6 mmol/l [100 mg/dl] of glucose will result in a 1.6 mmol/l rise in sodium concentration). The initial rise in serum sodium is not an indication to give hypotonic fluids, and the administration of 0.45% sodium chloride is indicated only if osmolality is not declining despite adequate positive fluid balance and appropriate insulin administration. Some have recommended that insulin be withheld until glucose has stopped dropping, with initial fluid administration alone to prevent a rapid fall in osmolality [117].

Older adults with DKA or HHS, as well as individuals with heart failure or end-stage kidney disease on dialysis, should be treated cautiously with smaller boluses of isotonic or crystalloid solutions (e.g., 250 ml boluses) and should undergo frequent assessment of haemodynamic status [131]. In such patients, the use of a standard fluid replacement protocol may be associated with treatment-related complications, including volume overload, need for mechanical ventilation and longer length of stay [131].

### Insulin

Insulin therapy is the cornerstone of DKA management and should be started as soon as possible after diagnosis. Short-acting insulin administered intravenously by continuous infusion is the preferred choice. Depending on the severity of the condition and the available facilities, this should be done using a fixed-rate intravenous insulin infusion started at 0.1 U kg^−1^ h^−1^ [1–3, 12, 132] or by a nurse-driven insulin infusion protocol with a variable rate for DKA [133]. In adults, treatment protocols recommend the initial administration of an insulin bolus (0.1 U/kg) (intravenously or intramuscularly) if a delay in obtaining venous access is anticipated to be followed by fixed-rate intravenous insulin infusion [12]. Once the blood glucose falls below 13.9 mmol/l (250 mg/dl), 5–10% dextrose should be added to the 0.9% saline infusion and the insulin infusion rate should be reduced to 0.05 U kg^−1^ h^−1^. Thereafter, intravenous insulin infusion should be adjusted to maintain glucose levels at approximately 11.1 mmol/l (200 mg/dl) and continued until the ketoacidosis is resolved [1–3].

In people on basal or basal-bolus insulin therapy before admission, this regimen can be continued at the usual dose and adjusted as needed. In those newly diagnosed, multidose insulin regimens with basal and prandial rapid-acting insulin analogues should be started after the resolution of DKA [1, 12]. Long-acting basal insulin should be initiated subcutaneously at 0.15–0.3 U/kg. This medication may be administered once daily or divided equally and administered twice daily. Rapid-acting insulin is added as needed, depending on nutritional intake and glucose levels.

The administration of basal insulin while on fixed-rate intravenous insulin infusion is advocated by many clinicians but avoided by others because of the risk of hypoglycaemia [134] or hypokalaemia [135]. Several studies have reported that the coadministration of a low dose (0.15–0.3 U/kg) of basal insulin during insulin infusion reduces time to DKA resolution, duration of insulin infusion [136, 137] and length of hospital stay [136] and prevents rebound hyperglycaemia, all without increased risk of hypoglycaemia [136, 138, 139].

Patients with uncomplicated mild or moderate DKA may be treated with subcutaneous rapid-acting insulin analogues [130, 138, 140]. Several randomised studies and a meta-analysis have reported that the administration of subcutaneous rapid-acting insulin analogues every 1–2 h is an effective alternative to intravenous infusion of short-acting insulin for people with mild or moderate DKA [138, 141, 142]. This treatment can be delivered in emergency departments and step-down units without the need for ICU care. A 2016 Cochrane review suggested that there were neither advantages nor disadvantages to using subcutaneous insulin over intravenous insulin when treating mild or moderate DKA [138]. Intramuscular rapid-acting insulin is also effective for treating DKA, but this route is more painful than subcutaneous injection and might increase the risk of bleeding for patients receiving anticoagulation therapy [1, 143]. The use of rapid-acting subcutaneous insulin analogues is not recommended for the treatment of severe and complicated DKA or with HHS.

Few studies have assessed the optimal insulin regimen in HHS. If the individual is already being treated with basal insulin, it should be continued at the usual dose and adjusted as needed. If HHS is present with no ketosis or with mild or moderate ketonaemia (blood β-hydroxybutyrate ≥1.0 to <3.0 mmol/l or urine ketones <2+) and without acidosis (pH ≥7.3 and bicarbonate ≥18 mmol/l), then a fixed-rate intravenous insulin infusion should be started at 0.05 U kg^−1^ h^−1^. If significant ketonaemia is present (i.e., β-hydroxybutyrate ≥3.0 mmol/l, ketonuria ≥2+, pH <7.30 or bicarbonate <18 mmol/l), which represents mixed DKA/HHS, then a fixed-rate intravenous insulin infusion should be started at 0.1 U kg^−1^ h^−1^ [117].

### Transition to maintenance insulin therapy

**Fig. 5:**
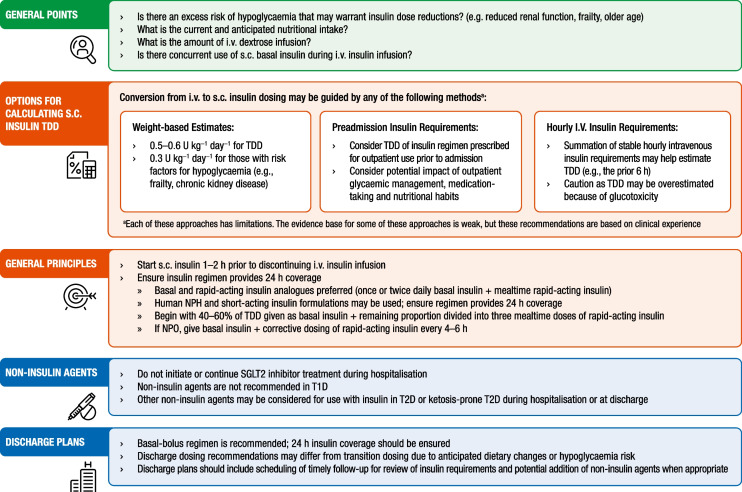
Transition to maintenance insulin administration in DKA. Calculation of the transition subcutaneous dose should account for hypoglycaemia risk factors and anticipated nutritional intake. Estimates can be made using a weight-based calculation or in those already on insulin, the preadmission insulin dose. Basal-bolus insulin is the preferred regimen and should be started 1–2 h before cessation of intravenous insulin. At discharge, dosing of basal-bolus insulin may change again considering hypoglycaemia risk. Follow-up plans should be in place to provide necessary support and training at discharge. NPO, *nil per os* (not by oral admnistration); T1D, type 1 diabetes; T2D, type 2 diabetes. This figure is available as part of a downloadable slideset

In the hospital, patients with DKA will eventually transition from intravenous to subcutaneous insulin, as illustrated in Fig. 5. To prevent the recurrence of hyperglycaemia or ketoacidosis during the transition period to subcutaneous insulin, it is important to allow an overlap of 1–2 h between the administration of subcutaneous insulin and the discontinuation of intravenous insulin. Patients with known diabetes may be given insulin at the dosage they were receiving before the admission. If there is concern for inadequate baseline insulin therapy (i.e., high HbA_1c_) or any potentially precipitating drug as a contributing factor to the DKA or HHS event, then the treatment regimen should be changed at discharge and not deferred to outpatient follow-up [1, 3, 12].

To transition from intravenous to subcutaneous insulin therapy, an estimation of the total daily insulin requirement is needed. This estimated total daily dose (TDD) of insulin may be calculated using several methods based on weight, preadmission insulin regimen or intravenous insulin requirements. However, each of these methods has limitations that must be considered when assessing overall insulin needs. First, a weight-based formula may be considered for TDD calculation using 0.5–0.6 U kg^−1^ day^−1^ in insulin-naive patients, with the understanding that body composition and/or insulin resistance may have an impact on this estimate [7, 12]. Similarly, for people with risk factors for hypoglycaemia, including kidney failure or frailty, a calculation using approximately 0.3 U kg^−1^ day^−1^ may be more appropriate. Second, consideration of the preadmission outpatient insulin regimen and HbA_1c_ levels may help guide transition dosing needs. However, it is necessary to understand how medication-taking behaviours and dietary factors may have influenced outpatient insulin dosing recommendations. Finally, TDD may be calculated by considering the hourly intravenous insulin infusion rate requirements, but with caution given the potential variation in insulin needs based on factors such as glucotoxicity, duration of treatment with intravenous insulin, concurrent dextrose infusion, medications associated with hyperglycaemia, and nutritional intake [144]. Once a TDD estimate has been determined, a multidose insulin regimen should be started, with basal insulin initiated at least 1–2 h before cessation of intravenous insulin infusion. Although first-generation basal analogues and NPH insulin are frequently administered once a day, greater flexibility and better coverage of basal insulin needs may be obtained if they are administered twice daily. The use of a basal-bolus insulin regimen with basal and rapid-acting insulin analogues has been proposed as a more physiological regimen and has been reported to reduce the rate of hypoglycaemia after transition from intravenous to subcutaneous insulin after resolution of DKA compared with human (i.e., short-acting and NPH) insulins [130]. Human insulin regimens may also be used, but proper dosing should ensure 24 h insulin coverage. There are no current studies on transitioning to ultra-long-acting insulin (e.g., degludec, glargine U300).

### Potassium

Despite experiencing a total-body potassium depletion of 3–6 mmol/kg due to long-standing osmotic diuresis, emesis and hyperaldosteronism [7], most patients with DKA present with normal or high serum potassium levels [10, 145]. This is because metabolic acidosis and insulin deficiency cause the movement of potassium from the intracellular to the extracellular compartment [146]. Insulin therapy, correction of acidosis, volume expansion and increased kaliuresis decrease serum potassium. Within 48 h of admission, potassium levels typically decline by 1–2 mmol/l during treatment of DKA, HHS and mixed DKA/HHS [24]. To prevent hypokalaemia, potassium replacement should be started after serum levels fall below 5.0 mmol/l to maintain a potassium level of 4–5 mmol/l [2, 12]. For most patients with DKA, 20–30 mmol of potassium per litre of intravenous fluid is sufficient to maintain a serum potassium concentration within the target range. Low-normal or low potassium levels (<3.5 mmol/l) are present on admission in 5–10% of patients with DKA [147]; in such cases, potassium replacement should begin at a rate of 10 mmol/h, and insulin therapy should be delayed until the potassium level increases to >3.5 mmol/l to avoid life-threatening arrhythmias and respiratory muscle weakness [147]. Severe hypokalaemia ≤2.5 mmol/l during treatment of DKA and HHS has been reported to be associated with a threefold increase in mortality [10]. To avoid hypokalaemia, we recommend measuring serum potassium 2 h after starting insulin administration and every 4 h thereafter until the resolution of DKA. Use of too low or too high doses of potassium compared with the recommended potassium replacement protocols in the management of DKA has been associated with longer hospital stays [148].

### Bicarbonate

Routine bicarbonate administration is not recommended. Intravenous fluid resuscitation and insulin administration are usually sufficient to resolve the metabolic acidosis of DKA [24, 149]. Several observational and randomised studies have reported that bicarbonate administration in DKA offers no advantage in improving cardiac or neurological outcomes or in the rate of recovery of hyperglycaemia and ketoacidosis [3, 12]. In addition, potential detrimental effects of bicarbonate therapy have been reported, such as an increased risk of hypokalaemia, decreased tissue oxygen uptake, cerebral oedema and development of paradoxical central nervous system acidosis [3]. However, because severe metabolic acidosis may lead to adverse vascular effects, bicarbonate administration should be considered if the acidosis is severe (i.e., pH <7.0) [146, 150]. If indicated, then 100 mmol of sodium bicarbonate (8.4% solution) in 400 ml of sterile water (an isotonic solution) can be given every 2 h to achieve a pH >7.0 [12].

### Phosphate

In DKA, there is a shift of phosphate from intracellular to extracellular fluid, with an excess urinary phosphate loss leading to hypophosphataemia [151]. Whole-body losses can be up to 1.0 mmol/kg; however, unless there is evidence of muscle weakness, such as respiratory or cardiac compromise with the phosphate <1.0 mmol/l, routine administration of phosphate is not indicated. Several prospective randomised studies have failed to show any beneficial effect of phosphate replacement on the clinical outcome of DKA [3, 152], and excessively rapid phosphate replacement may precipitate hypocalcaemia [152]. When necessary, 20–30 mmol of potassium phosphate can be added to replacement fluids. There is scarce data on phosphate deficiency or the effects of phosphate replacement in HHS, so we recommend a similar approach to phosphorus replacement.

### Criteria for resolution of DKA and HHS

Resolution of DKA is defined as achieving plasma ketone <0.6 mmol/l and venous pH ≥7.3 or bicarbonate ≥18 mmol/l [2]. Ideally, the blood glucose concentration should also be <11.1 mmol/l (200 mg/dl). The anion gap should not be used as a criterion, as it may be misleading because of the presence of hyperchloraemic metabolic acidosis caused by large volumes of 0.9% sodium chloride solution. Because β-hydroxybutyrate is converted into acetoacetate as the acidosis improves, urinary ketone measurement should be avoided as a criterion of DKA resolution.

While there is no consensus on the definition for resolution of HHS, we consider HHS to be resolved when the measured or calculated serum osmolality falls to <300 mOsm/kg, hyperglycaemia has been corrected, urine output is >0.5 ml kg^−1^ h^−1^, cognitive status has improved and the blood glucose is <13.9 mmol/l (250 mg/dl) [12, 117].

## Section 5. What are complications during treatment?

Table 3 describes current evidence, risks and mitigation strategies of the most important complications of treating acute hyperglycaemic crises in adults, including hypoglycaemia, hypokalaemia, normal anion gap metabolic acidosis, thrombosis, cerebral oedema, osmotic demyelination syndrome and acute kidney injury.

**Table 3:** Complications during treatment of DKA and HHS

Complication	Evidence	Risk	Mitigation
Hypoglycaemia [10, 24]	• Hypoglycaemia is a common complication encountered in the treatment of DKA. • In studies of DKA treatment, the risk of hypoglycaemia (<3.9 mmol/l [70 mg/dl]) varied between 16% and 28%, with severe hypoglycaemia (<2.2 mmol/l [40 mg/dl]) occurring in 2% of cases.	• Hypoglycaemia (<2.2 mmol/l [40 mg/dl]) during treatment was associated with a 4.8-fold increase in mortality (adjusted OR 4 [95% CI 1.4, 16.8]).	• Frequent blood glucose monitoring (every 1–2 h) is mandatory to recognise hypoglycaemia. • When glucose levels are reduced to <13.9 mmol/l (250 mg/dl), it is advised to reduce the insulin infusion rate to 0.05 U kg^−1^ h^−1^ and replacement fluids should be modified to contain 5–10% dextrose to prevent hypoglycaemia.
Hypokalaemia [24]	• Hypokalaemia is a common complication owing to intracellular shift of potassium following insulin treatment. • Hypokalaemia (<3.5 mmol/l) occurred in ∼55% of DKA and 51% of HHS patients. • Severe hypokalaemia <2.5 mmol/l occurs in 16% of people with DKA and 9% of people with HHS.	• Severe hypokalaemia ≤2.5 mmol/l was associated with increased inpatient mortality (adjusted OR 4.9 [95% CI 1.3, 18.8]).	• Potassium should be carefully monitored every 4 h during treatment. • Potassium replacement should be added to fluid resuscitation.
Normal anion gap metabolic acidosis [173, 174]	• Hyperchloraemic non-anion gap acidosis may be seen during the recovery phase of DKA, but the risk is unknown. It is likely to be caused by loss of keto-anions, which are metabolised to bicarbonate, and excess fluid infusion of chloride-containing fluids during treatment.	• Observed during the recovery phase of DKA, it is self-limiting with few clinical consequences.	• There is some evidence that hyperchloraemic acidosis occurs less frequently with balanced electrolyte solutions and when slower saline infusion is administered.
Thrombosis [43, 175, 176]	• Both DKA and HHS, but especially HHS, are thought to be prothrombotic states. • There is evidence that clot microstructure may be altered in people with acidosis and dehydration, but this is reversible.	• Although case series highlight the risk of venous and arterial thromboembolism in HHS, a nationwide Taiwanese study examining the risk of venous thromboembolism in people with HHS vs those hospitalised without HHS found similar rates.	• Currently, unless thrombosis is suspected, prophylactic dose low-molecular-weight heparin should be used to mitigate the risk of thrombosis.
Cerebral oedema [3, 177]	• Cerebral oedema is rare in adults. The underlying cause is not fully understood but may reflect osmotic changes, hypoperfusion and/or inflammatory responses. • In adult patients with HHS and DKA, rapid shifts in osmolarity may also be associated with cerebral oedema thought to occur in <0.1% of events.	• Cerebral oedema is a serious complication with a reported mortality of ∼30% compared with those without oedema. • Cerebral oedema may be subclinical and visible only on imaging studies.	• Recognising potential risk factors and being alerted to changes in mental status is advised, with a low threshold for brain imaging. • Mannitol infusion and mechanical ventilation are suggested for treatment of cerebral oedema. • In adults with HHS, a slow rate for correction of hyperosmolarity is indicated.
Osmotic demyelination syndrome [117, 178]	• Previously known as central pontine myelinolysis, osmotic demyelination syndrome can occur with rapid correction of hyponatraemia. The incidence is unclear.	• The risk is specifically associated with rapid correction of hyponatraemia. • May complicate treatment of adults with HHS where hyperosmolar patients may be relatively hyponatraemic.	• In patients with HHS, the fall in serum osmolarity should be corrected with 0.9% saline solution. • The fall in serum osmolality should be between 3.0 and 8.0 mOsm kg^−1^ h^−1^.
Acute kidney injury [179, 180]	• Using RIFLE criteria, 50% of adult patients admitted with DKA and HHS have acute kidney injury.	• Acute kidney injury is more common in older adults, those with higher osmolarity and those with higher admission glucose levels.	• Acute kidney injury usually resolves with rehydration. • Monitoring renal function daily is recommended.

RIFLE, risk, injury, failure, loss

## Section 6. What are the recommended management strategies for special populations?

Table 4 highlights some important considerations regarding DKA and HHS in special populations. These conditions or scenarios include frail older adults, individuals receiving SGLT2 inhibitor therapy, end-stage kidney disease requiring dialysis, pregnancy and COVID-19 infection.

**Table 4:** Features of DKA and HHS occurring in special populations

Special population	Clinical characteristics and presentation	Diagnostic considerations	Specific management considerations	Future care considerations
Frail or older adults [181]	• High rate of preexisting comorbidities. • High risk for hospital mortality, prolonged hospitalisation and DKA recurrences.	• Isolated HHS and mixed DKA/HHS occur more frequently than DKA. • Evaluate for specific precipitating factors and concurrent diagnoses (cardiovascular events, infection, medications).	• Fluid resuscitation and rate of fluid replacement need to account for comorbidities and acute precipitating events. • Address polypharmacy.	• Assessment of cognitive and functional status, including capacity for self-management. • Continued management of comorbidities and risk factors for DKA/HHS recurrence.
SGLT2 inhibitor [91, 93, 103, 182]	• May be spontaneous or preceded by insulin dose reduction or insulin omission, prolonged fasting or acute illness. • May be prevented using specific ‘sick day rules.’	• May present with near-normal glucose concentrations or euglycaemic DKA (glucose <11.1 mmol/l [200 mg/dl]).	• Acute management as for ‘general’ DKA. In euglycaemic DKA, 5–10% dextrose should be added to intravenous fluid or started at the same time as the 0.9% sodium chloride. • SGLT2 inhibitors should be stopped on admission.	• SGLT2 inhibitor therapy is not recommended for patients with T1D. • In patients with T2D, because of the lack of safety data, initiation or continuation of SGLT2 inhibitor therapy after DKA resolution is not routinely recommended.
End-stage kidney disease [2, 183]	• About 4% of patients with diabetes and end-stage kidney disease experienced DKA/HHS. • May present with fluid overload. High preexisting comorbidity burden with increased risk of mortality.	• Patients with end-stage kidney disease usually present with greater hyperglycaemia, more frequent hyponatraemia, higher osmolality, hyperkalaemia, and lower ketone concentrations of β-hydroxybutyrate compared with patients without end-stage kidney disease.	• Careful fluid administration and potassium replacement are needed. • Greater risk of cardiac co-complications.	• Holistic multidisciplinary care and aggressive multiple risk factor intervention is necessary. • Closer glucose and ketone monitoring is necessary.
Pregnancy [160, 184]	• Up to 2% of pregnancies with pregestational diabetes develop DKA. • Most cases occur with preexisting T1D. • The incidence of DKA in gestational diabetes is low (<0.1%).	• Euglycaemic DKA (glucose <11.1 mmol/l [200 mg/dl]) may occur. • Mixed acid–base disturbances may occur with hyperemesis, making the diagnosis challenging.	• The significant feto-maternal risk requires immediate expert senior medical and obstetric intervention. • Ideally patients should be cared for in delivery suites or high-dependency units.	• Management guidelines in the emergency department or obstetric unit should include sections on the management of DKA in pregnancy as well as sick day rules.
COVID-19 [79, 185]	• Higher frequency of DKA during the COVID-19 pandemic. • At-risk groups are adults with preexisting T2D. • High risk for complications, need for ICU care, longer hospital stays, and mortality.	• Usual diagnostic criteria. • Higher frequency of mixed DKA/HHS especially in older adults.	• Treatment with high-dose steroids requires higher-dose insulin to treat refractory ketonaemia. • In newly diagnosed individuals presenting with diabetes in DKA, diabetes phenotyping may be helpful.	• Discharge on insulin treatment with careful follow-up.

T1D, type 1 diabetes; T2D, type 2 diabetes

## Section 7. How can DKA and HHS be prevented?

Key issues at the time of hospital discharge include transitions of care, therapeutic inertia, the risk of hypoglycaemia and prevention of recurrent severe hyperglycaemic events. In US nationwide studies, up to 22% of people admitted with DKA had at least one readmission within 30 days or the same calendar year [25, 153]. Among those readmitted within 30 days, 40.8% represented recurrent DKA episodes, with approximately 50% being readmitted within 2 weeks [25]. Among those readmitted within the same calendar year, 86% and 14% had 1–3 and ≥4 readmissions for DKA, respectively [153]. Assessment of precipitating and contributing causes of DKA admission and close follow-up within 2–4 weeks after discharge may reduce recurrent DKA [154]. For example, the Novel Interventions in Children’s Healthcare programme supports families with children who have had multiple admissions for recurrent DKA [154, 155]. Similarly, close observation, early detection of symptoms and timely medical care help prevent HHS in older adults [154]. Presence of mental health disorders and SDOH need to be assessed on admission and before discharge. Extensive evidence indicates that mental health conditions—particularly eating disorders, depression or schizophrenia—are independent risk factors for poor glycaemic control and DKA [156]. Thus, regular screening of people with diabetes for psychological and behavioural disorders should be implemented in clinical practice.

Socioeconomic disadvantage is a major risk factor for DKA and HHS. Several indicators of socioeconomic disadvantage have been associated with an increased risk of hyperglycaemic crises. These include low income, homelessness, lack of health insurance or underinsurance, food insecurity and low educational attainment [59]. In a recent study, people from an area with the lowest income quartile had a 46% increase in the odds of four or more DKA readmissions in a given calendar year, while a patient with Medicare insurance had over a threefold increased odds of this outcome compared with those with private insurance [59]. In the USA, policy solutions such as increasing access to health insurance, affordable insulin, medical care, nutritious food and housing would be expected to reduce the incidence of DKA [157].

Before discharge, all individuals admitted with DKA or HHS should be offered appropriate education focused on both the current event and overall diabetes management. Patient education—especially structured education that includes problem-solving—is effective at reducing DKA admissions [158]. Participation in a structured diabetes education programme leads to a substantial risk reduction for DKA and HHS [156]. In patients with recurrent DKA, up to 75% of the admissions have been attributed to insufficient use of insulin therapy (i.e., missed insulin doses) as the immediate contributing factor [48]. Omission or insufficient use of insulin therapy is a major cause of DKA admissions and readmissions [159]. Thus, education on insulin administration and ‘sick day advice’ must be provided or reinforced. Upon discharge, patients should receive an adequate supply of insulin and diabetes-durable medical equipment (i.e., glucose monitoring and insulin administration devices) as well as contact information for healthcare professionals who can assist in managing future episodes of high blood glucose and ketone concentrations. For individuals with poor access to insulin, the social service department should be consulted to address these barriers to optimal self-management.

Education should include reviewing injection techniques (including sites), glucose monitoring, and urine or blood ketone testing [160]. Each patient and their family need to review the appropriate glucose and ketone monitoring and when to call for assistance. Home measurement of capillary blood and serum ketones helps to identify impending DKA [156]. Unfortunately, the rate of appropriate ketone monitoring, especially in adults, is low among people with diabetes [158, 161].

The ADA–EASD consensus report on type 1 diabetes recommends CGM as the monitoring method of choice for most people with type 1 diabetes [162]. CGM is superior to capillary blood glucose monitoring for improving glycaemic patterns among insulin-treated patients with type 1 diabetes and type 2 diabetes, especially those with out-of-range glucose levels. Results from a nationwide study in France reported that access to a CGM system was associated with a subsequent decrease in the rate of DKA hospitalisations by 53% and by 47% in type 1 diabetes and type 2 diabetes, respectively [163]. These results were observed both in patients treated with multidose insulin and in those treated with continuous insulin infusion (pump) therapy [164] Although CGM has not been approved for use in hospitalised patients with diabetes or with DKA, real-time or intermittently scanned CGM should be offered to people admitted with DKA after hospital discharge [165].

In individuals with multiple episodes of DKA, intensified and multidisciplinary approaches such as psychological interventions, peer support, individual coaching, and behavioural family systems therapy have been reported to reduce DKA risk [156]. In addition, the use of telemedicine and digital communication methods, as well as the provision of a 24 h emergency call service that offers medical advice for symptoms of DKA or when blood glucose or ketone concentrations are high, may reduce the risk of DKA admissions [156].

## Section 8. What are the priority areas for future research?

To date, clinical recommendations for the management of DKA and HHS are largely based on consensus and opinion rather than rigorous outcomes research. Thus, large randomised controlled trials or robust observational studies conducted in generalisable settings and populations are needed to determine the best management options, including optimising the electrolyte content of intravenous fluids (0.9% sodium chloride vs crystalloid solutions) as well as the optimal rates and techniques for insulin administration [2]. Small case series and retrospective studies suggest worse outcomes in patients with HHS compared with those with isolated DKA and that mixed DKA and HHS have worse outcomes compared with isolated DKA or HHS [2, 10]. However, no prospective studies have determined the best treatment for HHS and the combination of DKA and HHS. Dhatariya et al reported that despite potassium replacement following protocol in the UK, 67% of patients had a potassium level <4 mmol/l within 24 h of presentation [24]. Similar findings were reported in Canada [166] and the USA [10], where approximately 50% of patients developed hypokalaemia (<3.5 mmol/l) despite 91% of them receiving potassium replacement. Additional studies are needed to determine the ideal potassium replacement regimen in this clinical setting.

A high ketone concentration is the hallmark of DKA, with a consensus among clinical guidelines that a concentration ≥3 mmol/l correlates with acid–base parameters and severity of acidosis with >90% sensitivity and specificity for a diagnosis of DKA [117]. β-Hydroxybutyrate measurement can be performed as a laboratory test or using hydroxybutyrate and the nitroprusside methods. POCT of blood β-hydroxybutyrate is easy to perform and has advantages over laboratory measurement, although safeguards about staff training and instrument performance need to be in place [108]. Three areas of research interest include the use of real-time CGM at the time of hospital discharge [167], continuous interstitial ketone monitoring in the hospital and at home in high-risk individuals [168], and transitioning to ultra-long-acting insulin after resolution of DKA and HHS.

Because SDOH and structural barriers to accessing care are known drivers of susceptibility to hyperglycaemic crises, it is imperative to develop, implement and rigorously evaluate clinical, public health and policy interventions to prevent these events. Interventions by community health workers and community paramedics and even peer support interventions have been implemented to improve diabetes management, but these programmes have not been examined for impact on DKA or HHS. While prescribing healthy food can lead to substantial improvements in glucose levels, the impact of such interventions on hyperglycaemic crises is unknown. More information is needed about how to encourage behaviour that will lead to avoidance of DKA, especially in people with a history of recurrent episodes. Prospective studies focused on high-risk individuals with mental health disorders, diabetes distress and depression are needed [69, 169].

Finally, it will be important to understand the impact of lowering insulin prices in the USA, where insulin rationing—defined as skipping insulin doses, using less insulin than prescribed or delaying the purchase of insulin to save money—has been reported in up to 20% of people treated with insulin [170]. Cost-related insulin rationing is most commonly reported in non-Hispanic Black, middle-income, and underinsured or uninsured populations [48, 171] and has been associated with increased risk of DKA. Insulin supply remains a challenge in low-income countries despite insulin being included on the World Health Organization's list of essential medications. Additionally, further research is needed to understand better and ultimately eliminate the disparities in DKA and HHS rates experienced by racial and ethnic minority communities [16, 172]. In the USA, these disparities exist independent of other confounding risk factors for hyperglycaemic crises. Data on racial and ethnic disparities in DKA and HHS rates outside the USA are scarce and need to be examined. Ultimately, these disparities may call for comprehensive structural solutions, including at the clinician, health system, payer, public health and public policy levels. Optimal management of DKA and HHS will require greater knowledge of the pathophysiological, clinical and social roots of these serious complications of diabetes.

### Supplementary Information

Below is the link to the electronic supplementary material.

Slideset of figures (PPTX 702 KB)

## Abbreviations

CGM: Continuous glucose monitoring
COVID-19: Coronavirus disease-2019
DKA: Diabetic ketoacidosis
HHS: Hyperglycaemic hyperosmolar state
ICU: Intensive care unit
POCT: Point-of-care testing
SDOH: Social determinants of health
SGLT2: Sodium–glucose cotransporter 2
TDD: Total daily dose
