# Supplementary material for: Hyperglycaemic crises in adults with diabetes: a consensus report
Source: Diabetologia. 2024 Jun 22;67(8):1455–79. doi: 10.1007/s00125-024-06183-8 (PMC11343900; doi:10.1007/s00125-024-06183-8)
Supplement: Supplementary file 1 — Slideset of figures (PPTX 702 KB) [file 125_2024_6183_MOESM1_ESM.pptx]

## Slide 1
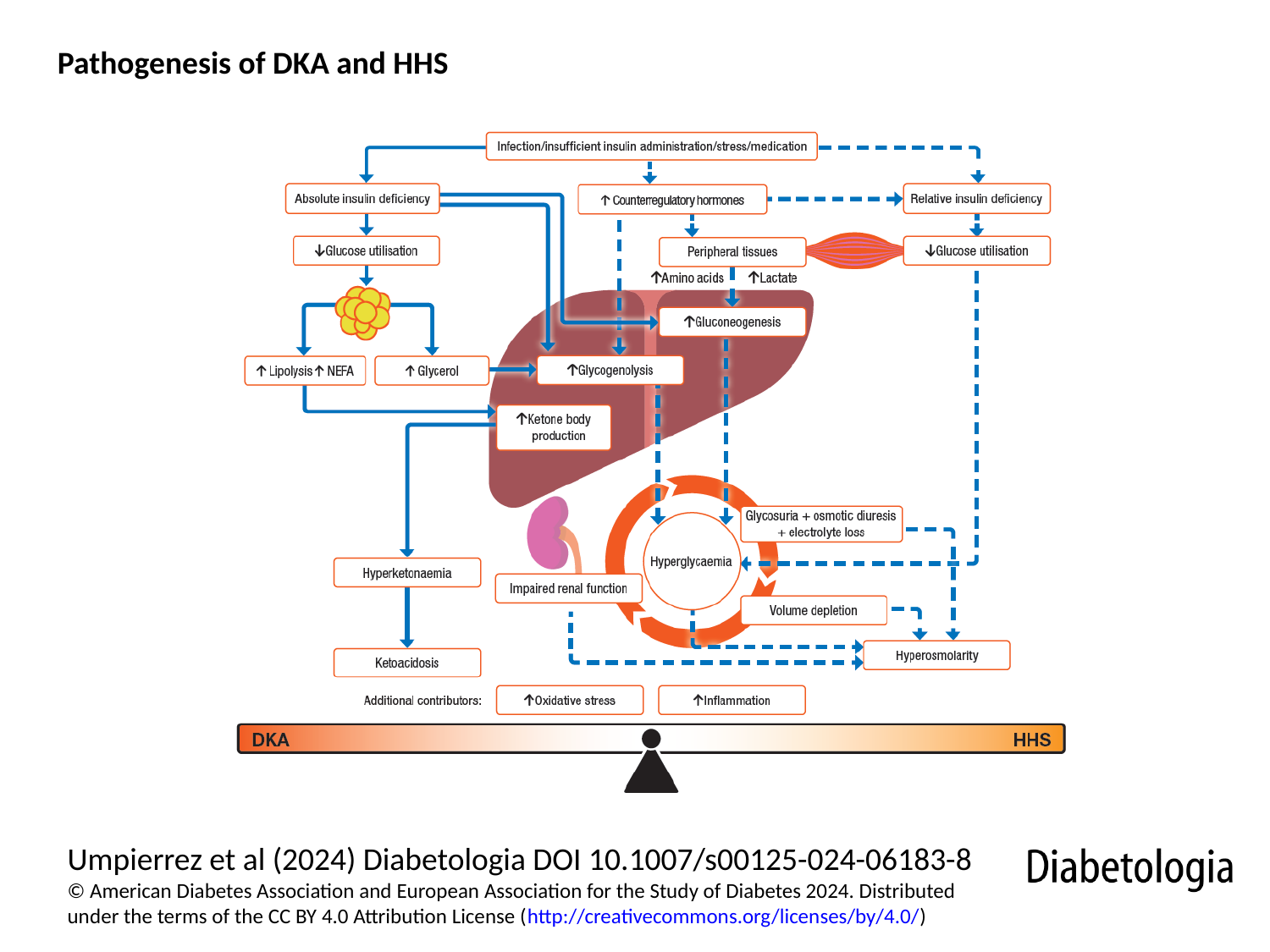

Pathogenesis of DKA and HHS
Umpierrez et al (2024) Diabetologia DOI 10.1007/s00125-024-06183-8
© American Diabetes Association and European Association for the Study of Diabetes 2024. Distributed under the terms of the CC BY 4.0 Attribution License (http://creativecommons.org/licenses/by/4.0/)

## Slide 2
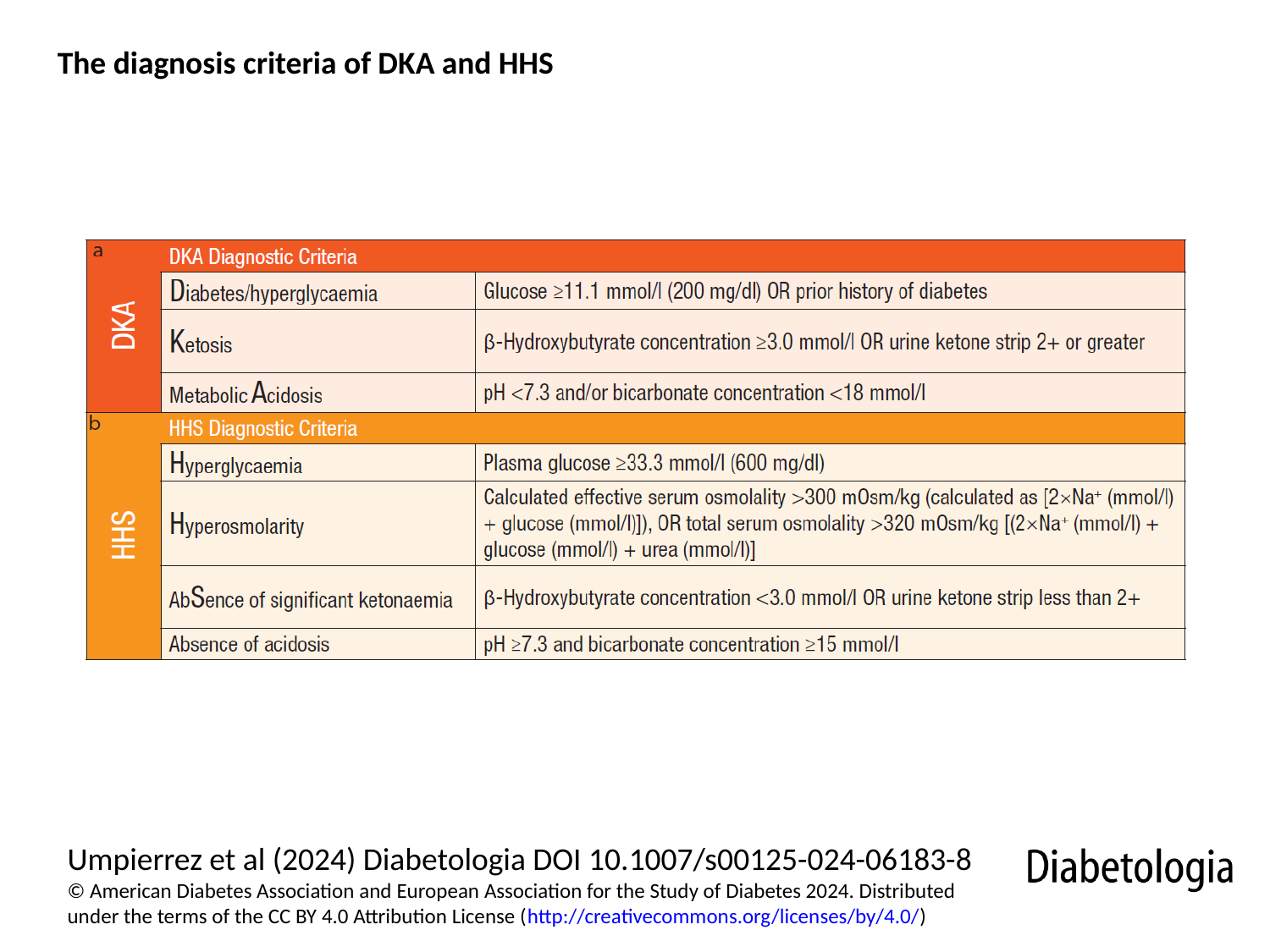

The diagnosis criteria of DKA and HHS
Umpierrez et al (2024) Diabetologia DOI 10.1007/s00125-024-06183-8
© American Diabetes Association and European Association for the Study of Diabetes 2024. Distributed under the terms of the CC BY 4.0 Attribution License (http://creativecommons.org/licenses/by/4.0/)

## Slide 3
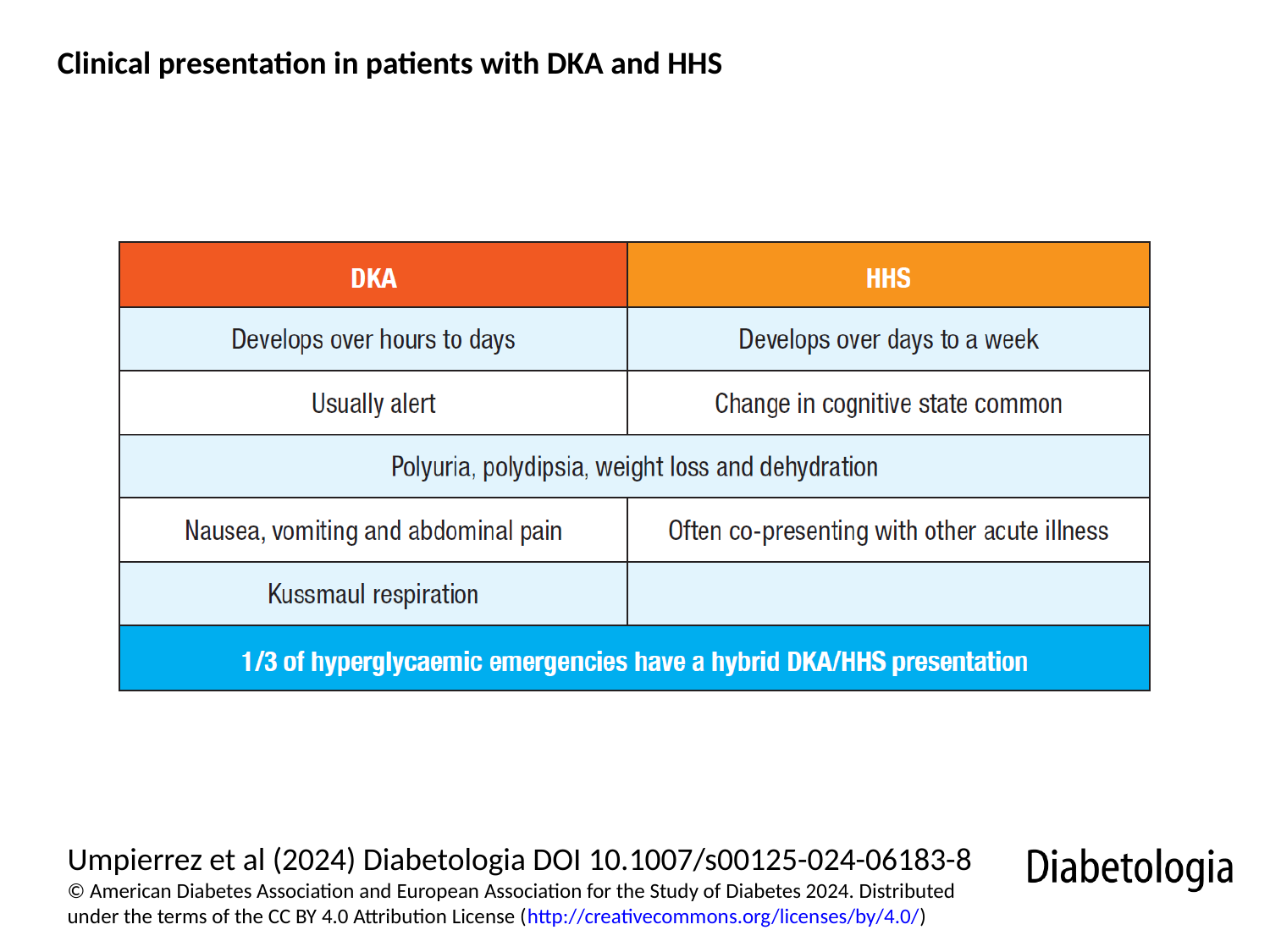

Clinical presentation in patients with DKA and HHS
Umpierrez et al (2024) Diabetologia DOI 10.1007/s00125-024-06183-8
© American Diabetes Association and European Association for the Study of Diabetes 2024. Distributed under the terms of the CC BY 4.0 Attribution License (http://creativecommons.org/licenses/by/4.0/)

## Slide 4
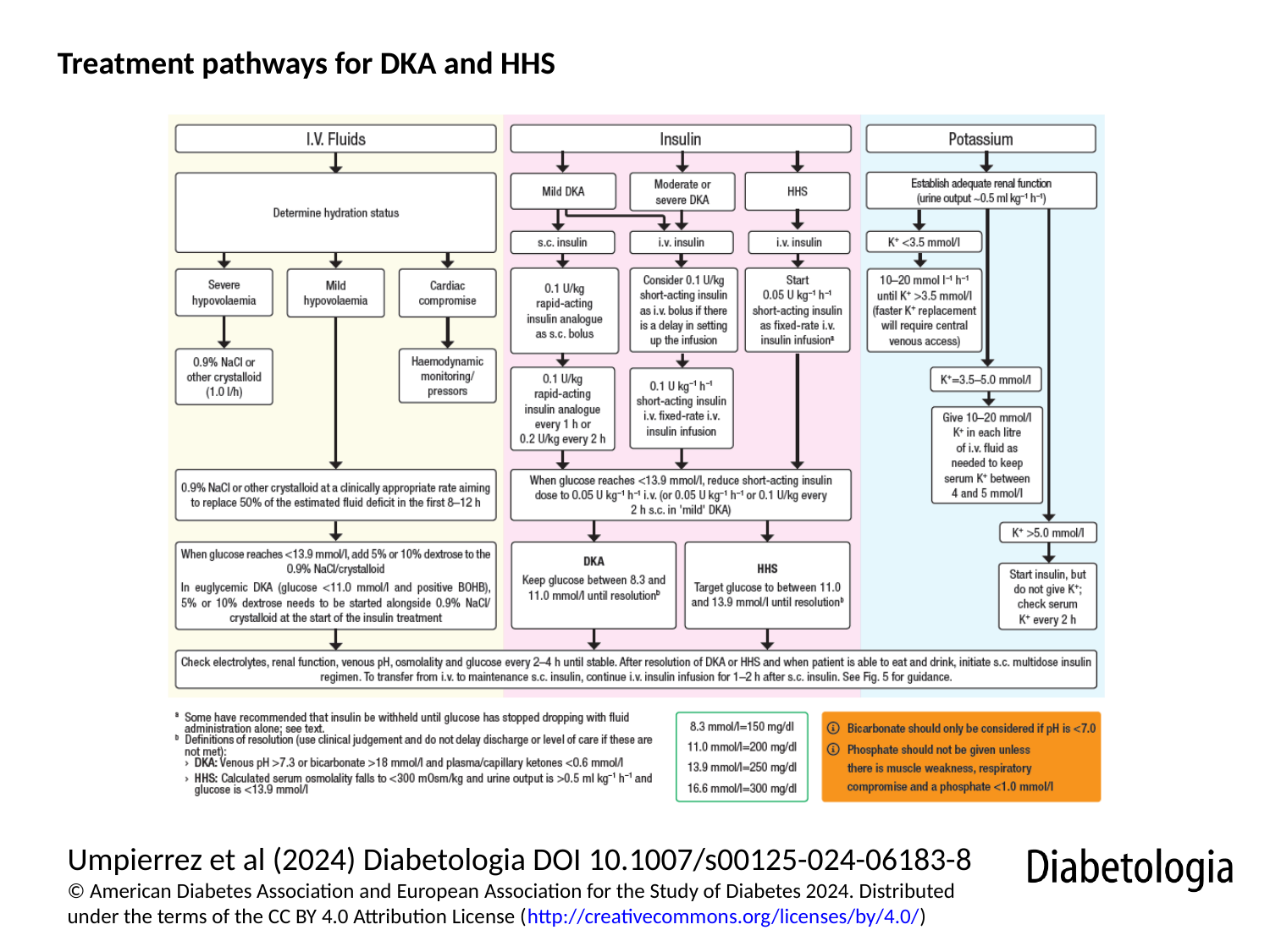

Treatment pathways for DKA and HHS
Umpierrez et al (2024) Diabetologia DOI 10.1007/s00125-024-06183-8
© American Diabetes Association and European Association for the Study of Diabetes 2024. Distributed under the terms of the CC BY 4.0 Attribution License (http://creativecommons.org/licenses/by/4.0/)

## Slide 5
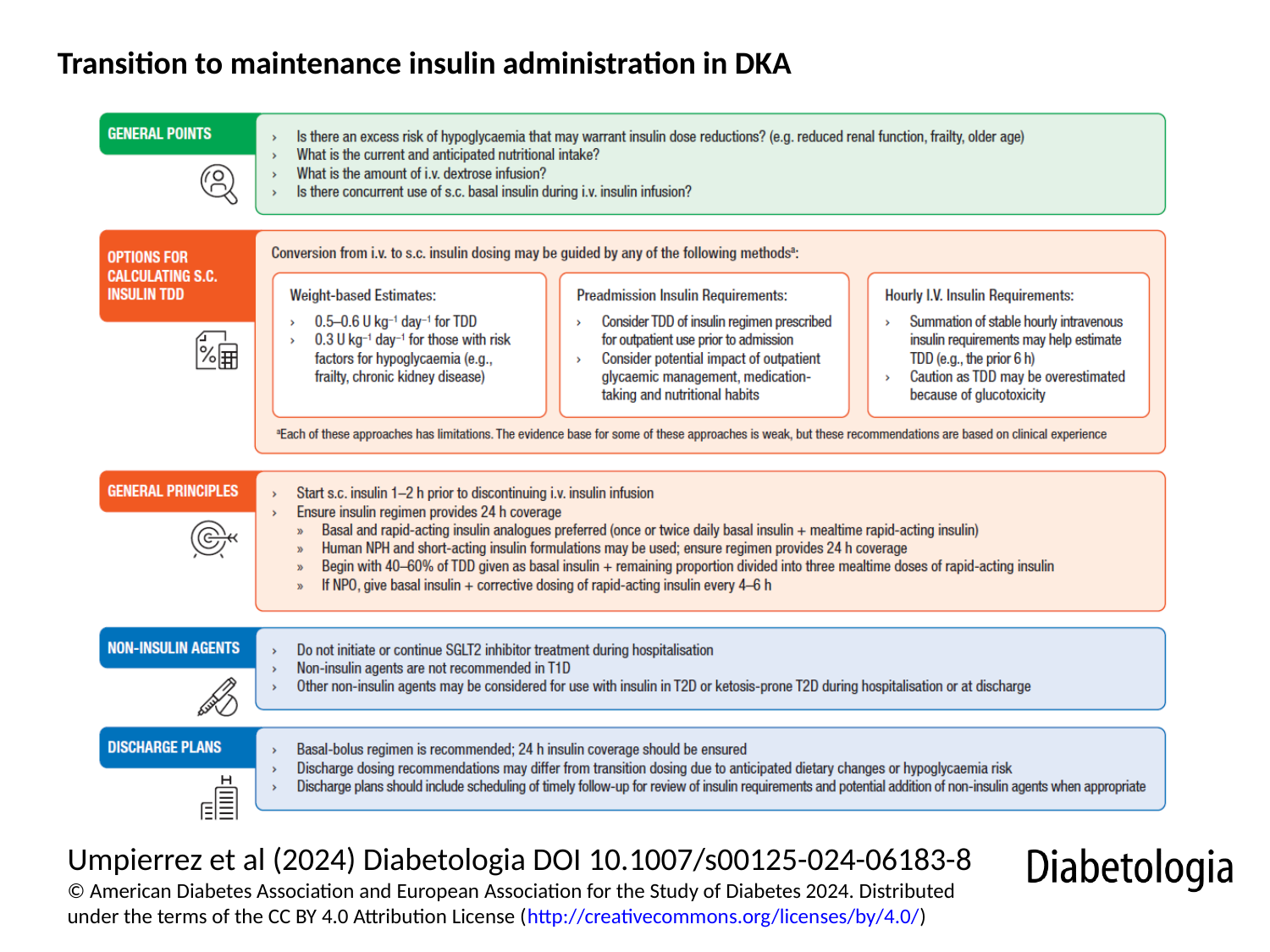

Transition to maintenance insulin administration in DKA
Umpierrez et al (2024) Diabetologia DOI 10.1007/s00125-024-06183-8
© American Diabetes Association and European Association for the Study of Diabetes 2024. Distributed under the terms of the CC BY 4.0 Attribution License (http://creativecommons.org/licenses/by/4.0/)
